# Gut flora connects obesity with pathological angiogenesis in the eye

**DOI:** 10.15252/emmm.201607165

**Published:** 2016-11-15

**Authors:** Rebecca Scholz, Thomas Langmann

**Affiliations:** ^1^ Laboratory for Experimental Immunology of the EyeDepartment of OphthalmologyUniversity of CologneCologneGermany

**Keywords:** Metabolism, Immunology, Neuroscience

## Abstract

Neovascular age‐related macular degeneration (nvAMD) can cause severe vision loss among the elderly. Genetic risk factors for AMD include several variants related to the immune system and lipid metabolism. Obesity is a well‐known predisposing factor for nvAMD but how this metabolic disorder modulates angiogenesis in the posterior eye segment was largely unknown. In this issue of *EMBO Molecular Medicine*, Andriessen *et al* (2016) show that high‐fat diet‐induced obesity causes dysbiosis in the gut that drives retinal inflammation and pathological angiogenesis in a mouse model of laser‐induced choroidal neovascularization (CNV).

AMD is a multifactorial aging disease with a strong genetic predisposition, for which smoking and diet, among others, are known environmental modifiers (Lim *et al*, 2012). The progression of early AMD with drusen deposits to the two main end stages of disease, namely geographic atrophy and CNV, is currently not well understood. All known genetic risk factors provide a framework for disease susceptibility but cannot explain the later stages of disease. However, there are epidemiological indications that unhealthy lifestyle and abdominal obesity increase the risk of progression to advanced forms of AMD (Adams *et al*, 2011).

There is a striking common denominator that connects aging, diet, and obesity, and this is the gut microbiota. Each healthy individual has a common set of co‐evolved gut colonizers (the core gut microbiota) that help us degrading otherwise indigestible food constituents (Backhed *et al*, 2005). The dominant microbiota species in the gut belong to the Bacteroidetes and Firmicutes divisions, and the presence of food leads to an enrichment of the Firmicutes species that increase the efficiency of intestinal fat absorption (Semova *et al*, 2012). Likewise, the proportion of Firmicutes is significantly higher in obese humans and mice (*ob/ob* mouse strain that carries a mutation in the gene for the satiety hormone leptin; Ley *et al*, 2005; Turnbaugh *et al*, 2006). These bacteria seem to help their host to get more calories from ingested food that will be used as energy. This phenotypic trait is transmissible as normal mice colonized with microbiota harvested from obese animals (“obese microbiota”) have a higher abundance of Firmicutes and significantly gain more weight compared to animals that received a “lean microbiota” (Turnbaugh *et al*, 2006). This implies that the gut microbiota changes rapidly upon dietary intervention and that the obesity‐associated gut microbiome primes the host for energy storage. Other forms of inadequate nutrition may occur during aging, and the microbiota of older people differs significantly from that of younger adults; such a loss of microbiota diversity is regarded as a biomarker of biological aging (O'Toole & Jeffery, 2015). Alterations in the composition of the gut microbiota, also termed dysbiosis, markedly affect the intestinal epithelial barrier and challenge the host immune system (Cerf‐Bensussan & Gaboriau‐Routhiau, 2010). A healthy diet and physiological microbiota are the basis of a balanced host–microbiota interaction that regulates intestinal and systemic immune homeostasis (Fig 1). Interestingly, microbiota also regulates immune cell function in the immune‐privileged central nervous system. Short‐chain fatty acids generated by fermentation activity of gut bacteria are mandatory for microglia to mature during development and respond to challenges in adult mice (Erny *et al*, 2015). Conditions of dysbiosis may lead to increased bacterial adherence to the mucus, damage of the epithelial barrier, and increased microbial‐associated molecular patterns that trigger systemic immunity via pro‐inflammatory cytokines and circulating immune cells (Fig 1).

**Figure 1:**
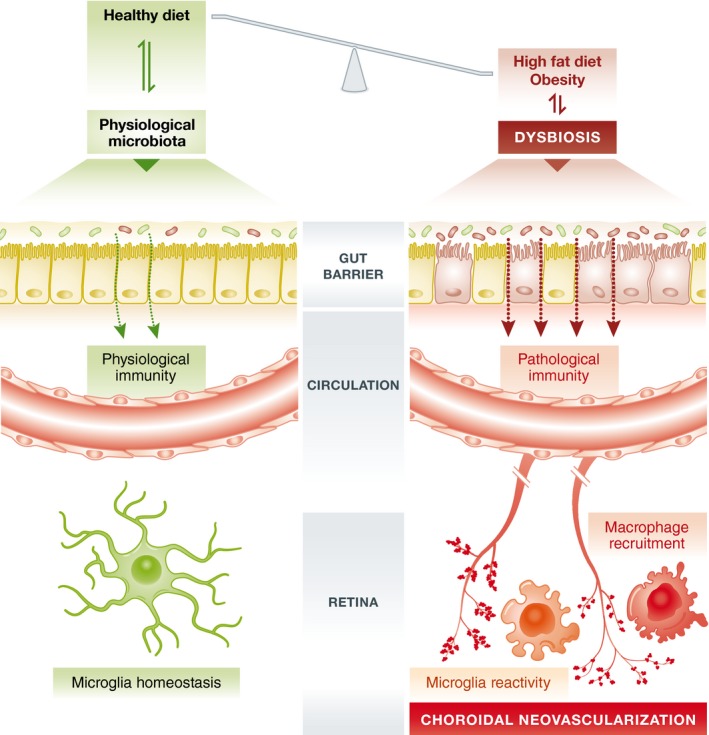
Host–microbiota interactions that regulate physiological and pathological immunity A healthy diet and physiological microbiota regulate physiological immunity that also controls microglia homeostasis in the central nervous system including the retina. Prolonged high‐fat diet and obesity can cause dysbiosis with impairment of the gut barrier and systemic inflammation. Microbial molecular pattern molecules and pro‐inflammatory cytokines appear in the systemic circulation and trigger immune responses in the retina. Microglia reactivity and inflammatory macrophage recruitment support the pro‐angiogenic milieu and finally lead to enhanced CNV.

The first indication that the intestinal microbiota could affect the distal ocular/retinal immune system came from studies in a mouse model of autoimmune uveitis, where retina‐specific T cells were activated by commensal microbiota‐derived antigens which further triggered autoimmunity in the retina (Horai *et al*, 2015). The experiments by Andriessen *et al* (2016) in this issue of *EMBO Molecular Medicine* now complement these findings and demonstrate that “obese microbiota” can challenge the retinal innate immune system to drive pathological angiogenesis. The authors first showed that high‐fat diet and concomitant weight gain make mice much more susceptible to experimental laser‐induced CNV. The research team suspected a potential role of gut microbes in this process and therefore administered a non‐gut permeable antibiotic orally to animals fed high‐fat diet. This treatment did not affect animal weight but prevented overt CNV, providing a direct connection between the gut microbiota and aberrant ocular angiogenesis. As previously shown for obese *ob*/*ob* mice (Ley *et al*, 2005; Turnbaugh *et al*, 2006), there was a shift from Bacteroidetes species toward more Firmicutes after mice received high‐fat diet and this dysbalance could be restored with antibiotics. The authors next performed transplantation experiments by oral gavage of fecal microbiota from healthy mice to obese animals and found significantly reduced pathological angiogenesis, indicating that dysbiosis and not weight gain is the key event in this process.

An important question was how gut dysbiosis affects distal CNV. Andriessen and colleagues answer this matter by carefully inspecting systemic and local immune parameters. They report that high‐fat diet increased gut permeability correlated with higher levels of circulating microbial‐associated pattern molecules. This triggered the systemic release of potent pro‐inflammatory mediators including interleukin‐6 (IL‐6), interleukin‐1b (IL‐1β), and tumor necrosis factor (TNF) which were also detected in the ocular choroid together with pro‐angiogenic vascular endothelial growth factor a (VEGFa). Resident microglia and recruited macrophages rapidly respond to these signals and establish a profound immune response in local lesion areas that sustains the CNV process (Fig 1). Interestingly, oral antibiotics could prevent microglia and macrophage reactivity and thereby limit CNV formation.

In conclusion, Andriessen *et al* (2016) provide a novel link between homeostasis of gut microbiota and ocular angiogenesis mediated via systemic and local immune processes. An important open question is how exactly resident microglia and macrophages sense the dysbiosis‐driven systemic inflammation. It is likely that the cells use their repertoire of pattern recognition receptors such as Toll‐like receptors and cytokine receptors but cell‐specific targeted deletion of individual receptors will be required to identify key players. Another interesting perspective is whether dietary interventions can help control dysbiosis, detrimental immunity, and CNV. The authors propose that modifying the gut microbiome could be a potential new therapeutic option for nvAMD patients. However, the gut flora is often unbalanced in the elderly and may hamper classical dietary interventions with vitamins and supplements. Therefore, the application of fiber‐rich prebiotics or specially designed probiotics that target metabolic overload may be more promising in preventing progression of early disease to late stage nvAMD.
